# Phytoremediation Assessment of *Mentha crispa L.* in Zinc-Contaminated Oxisols: Tolerance and Accumulation Dynamics

**DOI:** 10.1021/acs.jafc.4c08062

**Published:** 2024-12-31

**Authors:** Ana Flávia Bilmayer, Stephanie Locatelli, Martina Pomini, Thayná Francine Reis, Marcelo Hidemassa Anami, Edson Fontes de Oliveira, Robert Kowalik, Adriana Zemiani Challiol, Alessandra Furtado da Silva

**Affiliations:** † Graduate Program in Environmental Engineering (PPGEA), Federal University of Technology, Campus Londrina, Paraná 86036-370, Brazil; ‡ Department of Environmental Engineering, Federal University of Technology, Campus Londrina, Paraná 86036-370, Brazil; § Faculty of Environmental Engineering, Geomatics and Renewable Energy Kielce, Kielce University of Technology, 25-314 Kielce, Poland; ∥ Latin American Institute of Technology, Infrastructure, and Territory, Federal University of Latin American Integration, Foz do Iguaçu, Paraná 85870-650, Brazil

**Keywords:** growth analysis, tolerance, bioaccumulation factor, translocation factor, metal extraction rate

## Abstract

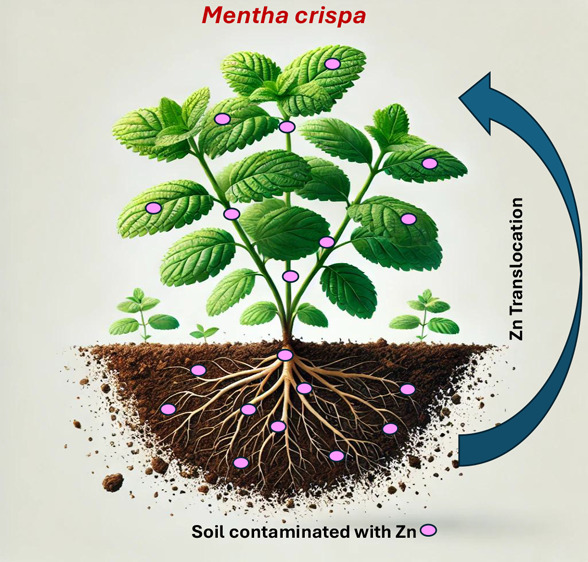

This study assessed the phytoremediation potential of *Mentha crispa L.* grown in Oxisol contaminated with varying zinc concentrations. *Mentha crispa* was cultivated in soil with Zn levels from 0 to 1920 mg kg^–1^. Growth parameters, Zn concentrations in plant parts, bioaccumulation, and translocation factors were measured. The results revealed that *Mentha crispa* exhibited a high tolerance to elevated Zn levels, accumulating up to 1875 mg kg^–1^ in its leaves and 2047 mg kg^–1^ in its roots. The bioaccumulation factor ranged from 1.2 to 4.5, and the translocation factor ranged from 1.2 to 2.7, indicating effective Zn uptake and distribution within the plant. The metal extraction rates varied across treatments, with the estimated time for Zn removal ranging from 12 to 34 years. These findings underscore the suitability of *Mentha crispa* as a candidate for phytoremediation of Zn-contaminated tropical soils, particularly Oxisols, which are characterized by high metal adsorption capacity. Additionally, its ability to produce essential oils enhances its viability for integrated environmental and economic applications.

## 1 Introduction

Soil contamination by heavy metals, particularly zinc (Zn), is a significant environmental issue caused by activities such as mining, agriculture, industrial processes, and improper waste disposal. Metals can accumulate in the environment, leading to mutagenic and carcinogenic effects that pose serious risks to both human health and ecosystems.^1−4^ Zn plays a dual role in the environment; as an essential micronutrient for plants, it is involved in critical biological processes, such as cell division, protein synthesis, and photosynthesis.^5^ However, when present in excess, Zn can have detrimental effects on plant health, causing symptoms such as growth inhibition, chlorosis, necrosis, reduced leaf area, impaired water absorption, and decreased photosynthetic rates, ultimately leading to nutritional imbalances.^5−8^

Anthropogenic sources of Zn contamination include mining and industrial activities, agricultural runoff, emissions from road traffic, and waste incineration.^9^ These sources contribute to the accumulation of Zn in soils, which can have long-term consequences for soil fertility and agricultural productivity. In humans, excessive Zn exposure through the food chain can lead to toxicity, manifesting in symptoms like nausea, vomiting, loss of appetite, abdominal cramps, and, in extreme cases, disruption of immune function and the metabolism of other essential minerals such as iron and copper.^10^ The urgency of addressing Zn contamination is particularly relevant in agricultural regions. According to the Brazilian National Environmental Council,^11^ Zn concentrations in soil above 450 mg kg^−1^ are considered investigation thresholds for agricultural areas, indicating a level at which potential harm to crops and human health must be assessed. Therefore, mitigating Zn pollution is crucial to maintaining ecosystem sustainability and ensuring the health of agricultural systems and communities that depend on them.

Oxisols, also known as Latossolos in tropical regions, are distinguished by their high capacity for metal adsorption, primarily due to the presence of iron and aluminum oxides in their composition.^12^ This characteristic makes them significantly different from other soil types, such as sandy or clayey soils, which have lower metal retention capacities. The high level of weathering that Oxisols undergo in tropical climates results in a soil profile rich in these oxides, enhancing their ability to bind and retain heavy metals like Zn. This makes Oxisols particularly relevant for phytoremediation studies, as their strong adsorption capacity helps to stabilize contaminants, reducing their mobility and potential for leaching into groundwater. This unique property positions Oxisols as an ideal candidate for examining plant–soil-metal interactions in tropical environmental remediation efforts.^13^

In this context, searching for developing technological solutions for removing metals from the soil is essential. Conventional methods (physicochemical) such as vitrification, soil washing, solidification, and stabilization are expensive and are only viable for small areas.^14^ Phytoremediation is an emerging and economically viable technique that uses plants to contain, isolate, remove, or reduce contaminant concentrations in soil and water.^15,16^ It is advantageous due to its low cost and environmental impact, improves soil fertility, restores ecosystem sustainability, and can be applied over larger areas.^7,17^

Aromatic plants have been studied for their environmental benefits and economic potential due to commercial exploitation by the pharmaceutical and food industries.^18^ Their secondary metabolites produce essential oils, cosmetics, and personal care products. Essential oil is a high-value commercial product obtained through steam-distillation, which minimizes the risk of metal contamination that remains in the plant residue.^6,19,20^

The most promising aromatic plants have been identified in the families *Poaceae*, *Lamiaceae*, *Asteraceae*, and *Geraniaceae*.^18^ Angelova et al.^21^ evaluated Lavender (*Lavandula vera* L.) for phytoremediation of soil contaminated with Cd, Pb, and Zn, finding it to be a hyperaccumulator for Pb and an accumulator for Cd and Zn. The metals were translocated to the aerial parts without affecting the plant’s development or the quality and quantity of the essential oil. Other studies have shown that Salvia species can be considered hyperaccumulators of metals^6,22−24^; Rosemary species as hyperaccumulators^25^ of Ni and phytostabilizers^26,27^ of various metals, Chamomile as phytostabilizers^28,29^ of Cd, and Basil as phytoextractors^30−32^ of Cd, Pb, and Zn.

Mint is notable for its highly vascularized roots, a characteristic that may enhance its potential as a phytoremediator.^33^ Various mint species have been studied in metal-contaminated soils, including *Mentha piperita*, which accumulated Cd, Cu, Cr, Mn, and Pb in its roots and Zn in its leaves without showing signs of Zn toxicity but with reduced biomass and essential oil production, though without changes in composition and quality.^34,35^ In *Mentha arvensis* and *citrata* species, biomass reduction and accumulation of Cd, Cr, Ni, and Pb in the roots and Cu, Mn, and Zn in the leaves were observed.^34−38^

Research on *Mentha crispa L.* for phytoremediation is still limited; however, some studies have demonstrated its potential for metal accumulation and tolerance. *Mentha crispa* can accumulate heavy metals, such as Pb and Cd, particularly in its roots, without significantly affecting plant growth or the composition of its essential oils.^39,40^ Although *Mentha* species have been studied for their tolerance and accumulation of heavy metals, their response to Zn contamination in tropical Oxisols remains unexplored. With high metal adsorption capacity due to iron and aluminum oxides, Oxisols present unique challenges and opportunities for phytoremediation. Investigating the suitability of *Mentha crispa* under these conditions is essential for advancing its application in environmental remediation and economic uses in tropical regions.

This study aims to evaluate the phytoremediation potential of *Mentha crispa L.* cultivated in Zn-contaminated tropical Oxisols. It focuses on the plant’s tolerance to varying Zn concentrations, analyzing growth parameters such as leaf count, shoot count, height, and biomass stability. Additionally, it investigates the efficiency of Zn accumulation in different plant tissues, quantified through bioaccumulation (BF) and translocation factors (TF), and evaluates the metal extraction rate (MER) under varying Zn concentrations. These objectives aim to provide a comprehensive understanding of the phytoremediation capabilities of *Mentha crispa*, emphasizing its suitability for sustainable remediation strategies in tropical soils, particularly Oxisols.

## 2 Materials and Methods

### 2.1 Chemical and Physical Soil Analysis

The soil was collected from the surface layer at a 0–20 cm depth, then crushed, sieved through a 2 mm mesh, and air-dried to obtain Fine Air-Dried Soil. Particle size analysis was performed using the sedimentation test with 0.1 M NaOH, agitation, and a hydrometer. Chemical analyses were conducted according to the Manual of Soil Analysis Methods by the Brazilian Agricultural Research Corporation.^41^ The parameters analyzed included cation exchange capacity (CEC), pH, organic matter (OM), total nitrogen (N), exchangeable bases, base saturation, phosphorus (P), potassium (K), magnesium (Mg), calcium (Ca), and aluminum (Al).

### 2.2 Adsorption Isotherms

Adsorption isotherms were applied using the Langmuir and Freundlich models to evaluate Zn adsorption in the soil. The experiment was conducted following the methodology used by Zemiani et al.^40^ Soil samples of 1 g at natural pH (5.5) were added to each Zn solution with concentrations ranging from 0 to 140 mg L^–1^, in the presence of 0.01 mol L^–1^ CaCl_2_. The resulting suspension had a final volume of 25 mL. Since pH influences metal adsorption onto soil particles, the pH values were adjusted close to the natural pH by adding small amounts of 0.1 mol L^–1^ HCl or NaOH solutions to the suspensions. Subsequently, the suspensions were agitated for 24 h, centrifuged at 3000 rpm for 10 min, and filtered.

The Zn concentration in each suspension’s filtrate (*C*_e_) was determined by flame atomic absorption spectrometry (SOLAAR S4, Thermo Scientific, USA). The concentration of Zn adsorbed by the soil particles (mg kg^–1^) was calculated by the difference between the total added concentration (*C*_total_) and the equilibrium filtrate concentration (*C*_e_), considering the suspension volume (*V*) of 25 mL and the soil mass (*m*) of 1 g, according to eq 1.
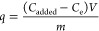
1Where *q* is the metal adsorbed per mass of soil (mg kg^–1^), *C*_total_ is the Zn total concentration added (mg L^–1^), *C*_e_ is the Zn concentration obtained from the filtrate (mg L^–1^), *V* is the slurry volume (0.025 L), and *m* is the mass of the soil (0.001 kg).

The Langmuir and Freundlich models^42^ were applied to the adsorption isotherms, *q* (mg kg^–1^) as a function of *C*_e_ (mg L^–1^), to estimate the maximum adsorption capacity (*q*_0_) of Zn in the soil. Equations 2 and 3 correspond to the Langmuir and Freundlich models, respectively.
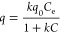
2
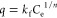
3Where *q* is the metal adsorbed per mass of soil (mg g^–1^), *k* is the constant related to bond energy (L mg^–1^), *q*_0_ is the maximum adsorption capacity (mg g^–1^), *C*_e_ is the concentration of metal in equilibrium (mg L^–1^), *k*_f_ is the constant of Freundlich, and *n* is the soil affinity parameter.

### 2.3 Soil Contamination with Zinc

In this experiment, the range of Zn concentrations applied to the soil for the cultivation of *Mentha crispa* was arbitrarily established using concentrations below and above 480 mg kg^–1^, which is close to the investigation threshold for agricultural areas of 450 mg kg^–1^ (CONAMA, 2009) and the maximum adsorption capacity (MAC) determined following the methodology described in Section 2.2. The Zn concentrations are presented in Table 1, where each concentration used (treatment, T_0_ to T_7_) was conducted with five replicates. Treatment T_0_, with no Zn added to the soil, served as the control.

**Table 1:** Zn Concentrations Were Applied to the Soil for the Cultivation of *Mentha crispa*^a^

treatment	Zn concentration in the soil, mg kg^–1^
T_0_	0.0 (as control)
T_1_	60
T_2_	80
T_3_	120
T_4_	240
T_5_	480 (close to MAC)
T_6_	960
T_7_	1920

a Each treatment was conducted with five replicates, and T_0_, with no Zn added, served as the control for the experiment.

The experiment was conducted in a greenhouse under natural light and temperature conditions (19.9 °C ± 6.3), with humidity levels maintained at 60%. Polypropylene pots, each containing 2 kg of soil, were used. After soil contamination with Zn, the pots were kept in the greenhouse for 30 days to reach equilibrium. Zinc acetate dihydrate (C_4_H_6_O_4_Zn·2H_2_O) was used as the contaminant to avoid anions like nitrate or chloride, which could act as nutrients and mask the effect of Zn on plant development.

### 2.4 Cultivation of *Mentha crispa* and Plant Growth Analysis

*Mentha crispa* seedlings were purchased commercially and initially cultivated in small plastic bags. After reaching approximately 10 cm in height, the seedlings were transplanted into the prepared soil as described in Section 2.3, with one seedling per pot. The experiment was conducted in a greenhouse for 105 days, with humidity maintained at 60% by weighing the pots and replacing the water lost through evaporation.^43^ Nitrogen, phosphorus, and potassium fertilizers were applied at the start of the experiment and again at 45 days for all treatments, including the control. The applied dosages were 47 mg L^–1^ of ammonium sulfate [(NH_4_)_2_SO_4_], 10 mg L^–1^ of potassium chloride (KCl), and 95 mg L^–1^ of monopotassium phosphate (KH_2_PO_4_), following the regional soil fertilization and liming manual.^44^

Throughout the development of *Mentha crispa*, daily inspections were conducted to record any visual changes in the plants compared to the control. For plant growth analysis, parameters such as height, number of shoots, and number of leaves were measured biweekly using a ruler (for height) and manual counting (for shoots and leaves). After the 105-day cultivation period, the plants were harvested and separated into three parts: roots, stems, and leaves. These parts were cleaned with ultrapure water (Purelab Option Q-7, Elga, UK). The roots underwent an additional cleaning step by immersion in a 10% v/v HCl solution (distilled, subboiling BSB-939-IR, Berghof, Germany) for 1 min to remove soil particles, followed by rinsing with ultrapure water. After cleaning, the plant parts were dried with paper towels and weighed to determine the biomass produced.^40^

### 2.5 Determination of Zn Concentration

The replicates of each plant part (roots, stems, and leaves) from each treatment were combined to form a composite sample to determine the average Zn concentration. The samples were dried in an oven with forced air circulation at 60 °C until a constant mass was achieved. Subsequently, the material was ground using a Willye-type knife mill, macerated, and sieved through a polyester mesh with a 75 μm opening.

The dried, macerated, and sieved samples were subjected to acid digestion in a digestion block. For each sample, 0.3 g was used in triplicate, with the addition of 4 mL of nitric acid (65% v/v, distilled, Subboiling BSB-939-IR, Berghof, Germany), and heated to 50 °C for 30 min. Subsequently, the temperature was increased to 120 °C for 90 min. After this period, the samples were cooled to room temperature, and 4 mL of hydrogen peroxide (30% v/v) was added, with the temperature raised to 120 °C for an additional 20 min.^45^ After cooling, the entire mixture was filtered and transferred to a polypropylene tube, with the final volume adjusted to 20 mL using ultrapure water.

A mass of 0.1 g of uncontaminated soil was weighed, and 2 mL of aqua regia (HCl 3:1) and 0.5 mL of concentrated HClO_4_ were used to determine the natural concentration of Zn in the soil. The mixture was subjected to a digestion block for 6 h at 90 °C.^46^ The material was then filtered and transferred to a polypropylene tube, with the final volume adjusted to 20 mL using ultrapure water. The determination of Zn concentration in the digested samples was performed using flame atomic absorption spectrometry (SOLAAR, Thermo Scientific, USA) with an air-acetylene flame, a hollow cathode lamp operating at 213.9 nm, deuterium background correction, and other parameters recommended by the manufacturer.

### 2.6 Bioaccumulation and Translocation Factors and Metal Extraction Rate

The potential for Zn absorption by *Mentha crispa* can be estimated through Bioaccumulation and Translocation Factors. The Bioaccumulation Factor (BF) is defined as the ratio of the plant’s metal concentration to the soil’s metal concentration, serving as an indicator of the plant’s ability to accumulate metal in its tissues. The Translocation Factor (TF) refers to the efficiency of the plant in accumulating the metal in its aerial parts, defined as the ratio of the metal concentration in the aerial parts to the metal concentration in the roots. The BF and TF can be calculated using eqs 4 and 5.^47^
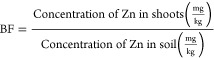
4
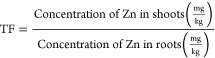
5

The Metal Extraction Rate (MER) expresses the plant’s ability to extract metal from the soil, considering its produced biomass and the decontaminated volume.^48,49^ It can be calculated using eq 6.
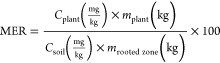
6Where *C*_plant_ is the metal concentration in the shoots (stem + leaves) of the plant, *m*_plant_ is the mass of the harvestable aboveground biomass produced in one harvest, *C*_soil_ is the metal concentration in the soil volume, and *m*_rooted zone_ is the mass of the soil volume rooted by the plant.

### 2.7 Data Analysis

Data on the growth of *Mentha crispa* over the experimental period, the Zn concentrations absorbed by the plant, and the bioaccumulation and translocation factors were analyzed using the means of replicates for each treatment. The results were subjected to analysis of variance (ANOVA), followed by the Scott-Knott test to compare means at a significance level of ρ < 0.05.

The assumptions of normality and homogeneity of variances were evaluated prior to ANOVA. The Shapiro-Wilk test showed that while some groups met the normality assumption (ρ > 0.05.), others did not (ρ ≤ 0.05). Levene’s test indicated nonhomogeneous variances (ρ = 0.0146). However, ANOVA was applied due to its robustness to mild violations of these assumptions, particularly in balanced experimental designs like this one. The Scott-Knott test was subsequently used to group means and identify significant differences. All analyses were conducted using RStudio software.

Canonical Discriminant Analysis (CDA) is a statistical technique used to determine the most critical variables in segregating two or more formed groups. CDA was performed to identify which plant variables (height, number of shoots, and number of leaves) were most important in differentiating between groups with different Zn concentrations and, therefore, which were most affected by Zn treatments in the soil (T_0_ to T_7_).^40^ These tests were processed using Statistica Release 7 software.

## 3 Results and Discussion

### 3.1 Physical and Chemical Properties of the Soil

The soil composition of clay, silt, and sand resulted in proportions of 73, 15, and 12%, respectively, classifying it as having a clayey texture (particle diameter less than 2 μm). The results of the chemical analyses are presented in Table 2.

**Table 2:** Soil Chemical Analysis

	unit	value
pH (H_2_O)		5.5
pH (CaCl_2_)		5.0
Organic matter	g dm^–3^	24.0
Total exchangeable bases	cmol_c_ kg^–1^	6.9
Base saturation	**%**	52.1
Cation-exchange capacity (CEC)	cmol_c_ kg^–1^	13.3
N (total)	g dm^–3^	1.2
P	mg kg^–1^	7.5
K	cmol_c_ kg^–1^	0.5
Mg	cmol_c_ kg^–1^	1.9
Ca	cmol_c_ kg^–1^	4.4
Al^3+^	cmol_c_ kg^–1^	0.00
Zn^2+^	mg kg^–1^	7.1

The obtained values of 24.02 g dm^–3^ and 13.3 cmol_c_ dm^–3^ for OM and CEC were considered moderate.^50^ Nascimento and Fontes^51^ observed that the clay content in the soil is a determining factor for adsorption due to its effect on CEC and its influence on OM content. CEC indicates the soil’s capacity to exchange ions of the same charge, and OM facilitates metal adsorption via complex formation, affecting the availability of metals to plants. Phosphorus (P), Potassium (K), and Nitrogen (N) are essential macronutrients for plant development, and the values obtained in this study (Table 2) were considered moderate.^52^

The percentage of exchangeable base saturation is an excellent indicator of soil fertility conditions. Eutrophic (fertile) soils have values ≥50%, while dystrophic (less fertile) soils have values below 50%.^53^ Based on chemical analyses, the soil was classified as Dystroferric Red Oxisol (LVdf), with eutrophic fertility, confirming the results described by Bognola et al. (2011).^77^

**Figure 1:**
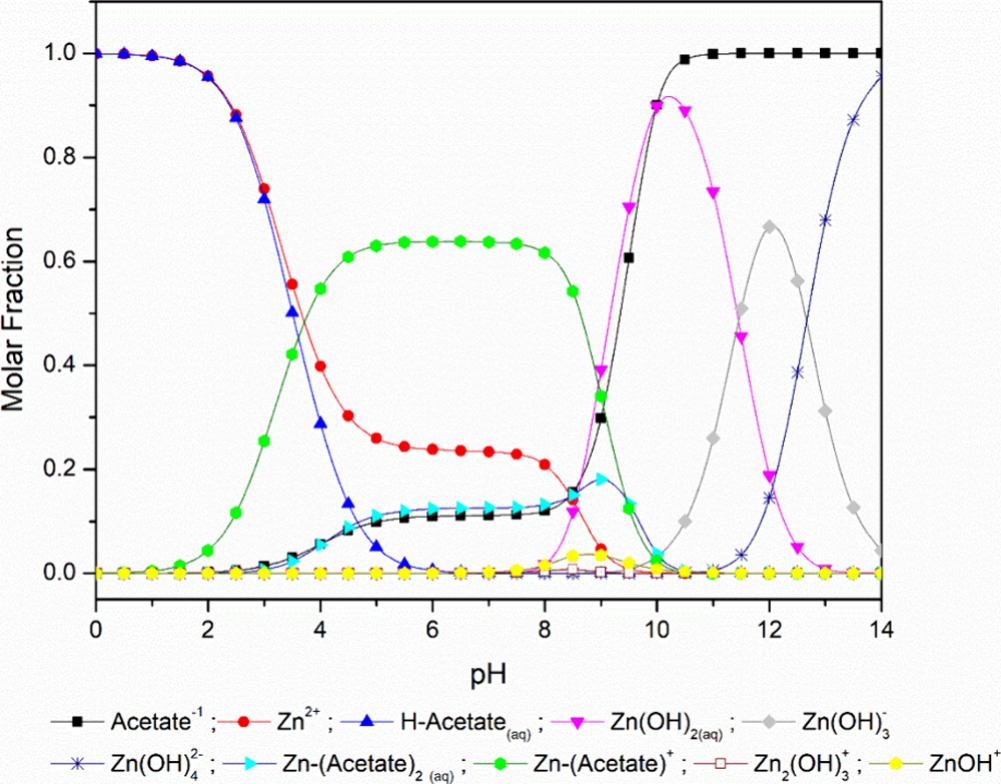
Distribution of aqueous Zn species as a function of pH, considering the Zn-acetate salt, using *Visual MINTEQ* modeling.

The pH values (Table 2) indicate a moderately acidic soil.^54^ Since pH is the most important factor in determining the metal species in soil,^55^ and considering the zinc salt (zinc acetate) used in the experiment, we can determine the distribution of molar fractions of Zn species as a function of pH (Figure 1) using the *Visual MINTEQ* software.^56^ As shown in Figure 1, the predominant Zn species found within the pH range of this study were *Zn*–*acetate*^+^ and *Zn*^2+^, which are soluble species and thus more available to plants. The hydrolyzed Zn species Zn (*Zn(OH)*_*2(aq*)_, *Zn*(*OH*)_3_^–^, *Zn*(*OH*)_4_^2–^) can be disregarded as their predominance occurs only at pH above 8. The protonated species *H*–*acetate*_*aq*_, without Zn, can also be neglected since its molar fraction increases at pH levels below 4.

### 3.2 Adsorption Isotherms

**Figure 2:**
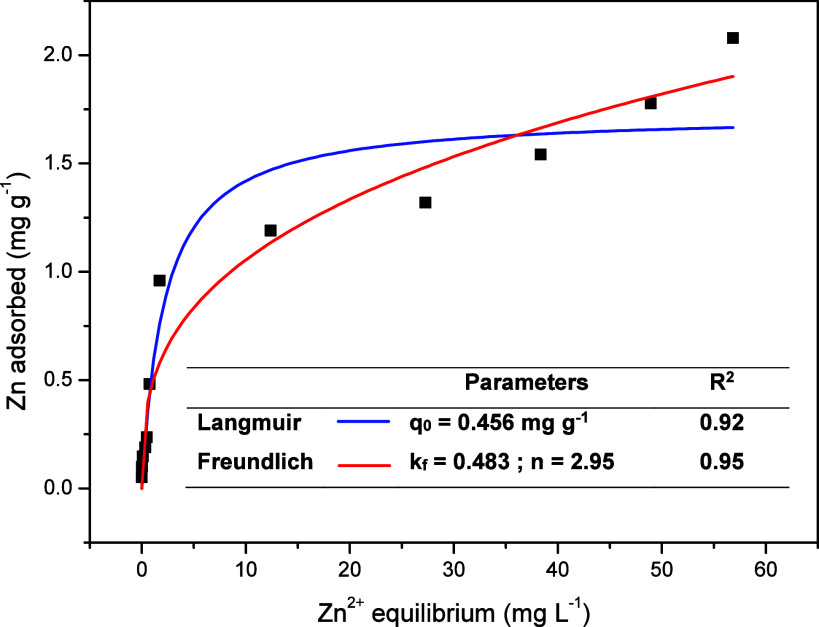
Langmuir and Freundlich’s isotherms applied to Zn adsorption in Oxisol.

The Langmuir and Freundlich models were applied to estimate the adsorption behavior of Zn in Oxisol and are illustrated in Figure 2. According to Figure 2, the Langmuir and Freundlich isotherms are similar at lower Zn concentrations. As Zn concentrations increase, the Freundlich model diverges from the Langmuir model and fits the experimental data better. Considering both models, the Freundlich model obtained the best fit, with a *k*_f_ of 0.483 and a determination coefficient (*R*^2^) of 0.95.

The maximum adsorption capacity (MAC) of Zn in Oxisol estimated by the Langmuir isotherm (*R*^2^ = 0.92) was 456 mg kg^–1^ (*q*_0_), comparable to the value of 483 mg kg^–1^ obtained for the same soil type by Casagrande et al.^57^ The MAC value obtained in this study was close to the investigation threshold for agricultural areas of 450 mg kg^–1^ and the value used in treatment T_5_ of 480 mg kg^–1^. This value indicates that treatments T_6_ and T_7_, with Zn concentrations in the soil exceeding the MAC value, had a higher fraction of free Zn species (*Zn*–*acetate*^+^ e *Zn*^2+^) that plants can readily absorb. In treatments T_1_ to T_5_, with values below and close to the MAC, most of the metal was adsorbed by soil particles, hindering the absorption process by plants.

### 3.3 Zn Effect on Plant Growth

**Figure 3:**
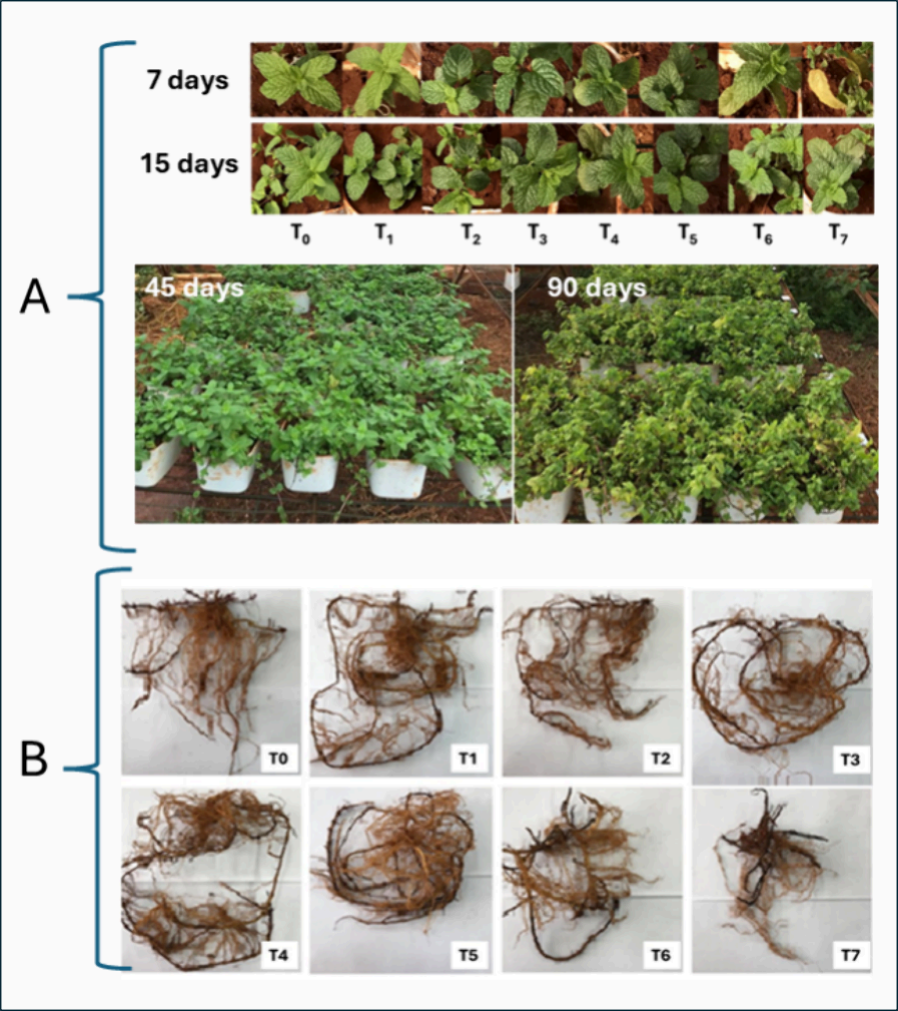
(A) *Mentha crispa* after 7, 15, 45, and 90 days of cultivation in Zn-contaminated soil with different concentrations (T_1_ to T_7_) and the control (T_0_), with one seedling per pot. (B) Image of the cleaned and dried roots after 105 days of growth, with treatments ranging from T_1_ to T_7_ and T_0_ as the control.

The first visual differences in *Mentha crispa* grown in Zn-contaminated Oxisol compared to the control (T_0_) were observed after the initial weeks (7 and 15 days) of the experiment and are shown in Figure 3A.

After 7 days, some leaves were yellowed in treatments T_6_ and T_7_, along with leaf drops in three replicates of treatment T_7_. Since the Zn concentrations in the soil for treatments T_6_ and T_7_ were above the MAC, the yellowing and leaf drop may be attributed to the excess Zn in the soil, reducing the efficiency of the photosynthesis system.^5,8,10,58^ After 15 days of cultivation, the plants in treatments T_6_ and T_7_ showed adaptation, with no signs of drying or leaf loss.

The visual differences between treatments diminished over time, as can be seen at 45 and 90 days (Figure 3A), indicating the adaptation of *Mentha crispa* to soil contaminated with high concentrations of Zn. After 105 days of the experiment, the plants were harvested and separated into roots, stems, and leaves. The image of the cleaned and dried roots is presented in Figure 3B. Darkening and, notably, a reduction in root length and hair were observed for treatments with the highest Zn concentrations (T_6_ and T_7_), which corroborates other studies on different plant species.^5,59,60^ High Zn concentrations in the soil can lead to reduced root growth by increasing the pectin content, which binds to the excess Zn in the cell wall, preventing its entry into the cytoplasm and its participation in essential metabolic and physiological processes.^5,61^ Reduced root growth diminishes the absorption of water and nutrients, potentially affecting plant development.^5^

The biomass produced by *Mentha crispa* can be seen in Table 3, which shows the average dry weights of roots, stems, and leaves obtained from the replicates for each treatment. The aboveground biomass (stem and leaf) produced by the plant in all treatments (T_1_ to T_7_) did not show a significant difference compared to the control treatment (T_0_), even at high Zn concentrations in the soil (1920 mg kg^–1^, T_7_).

**Table 3:** Average Dry Weights (g) of Roots, Stems, and Leaves of *Mentha crispa* Grown in Zn-Contaminated Oxisol (Treatments T_1_ to T_7_, and T_0_ as Control)^a^

treatment	roots	shoots	leaves
T_0_	6.7 ± 1.0^a^	2.3 ± 1.2^a^	3.0 ± 1.4^a^
T_1_	13.8 ± 8.0^ac^	4.7 ± 1.5^a^	3.9 ± 1.6^a^
T_2_	15.6 ± 7.6^ac^	3.9 ± 2.0^a^	3.3 ± 1.4^a^
T_3_	17.4 ± 7.1^abc^	4.3 ± 1.9^a^	4.5 ± 2.2^a^
T_4_	30.1 + 5.4^bc^	4.4 ± 1.9^a^	4.6 ± 2.4^a^
T_5_	23.9 ± 9.4^c^	4.5 ± 1.9^a^	4.9 ± 2.4^a^
T_6_	17.0 ± 5.0^abc^	5.7 ± 2.2^a^	4.4 ± 2.1^a^
T_7_	6.5 ± 4.6^a^	6.9 ± 2.4^a^	4.5 ± 1.9^a^

a Tukey test, *p* < 0.05.

Considering the roots, the masses were generally greater with the increase in Zn concentration in the soil, particularly in treatments T_4_ and T_5_, compared to the control treatment T_0_. The smaller and similar masses obtained for treatments T_0_ and T_7_ may indicate Zn deficiency in the soil for T_0_ and Zn excess in T_7_, causing toxicity.

**Figure 4:**
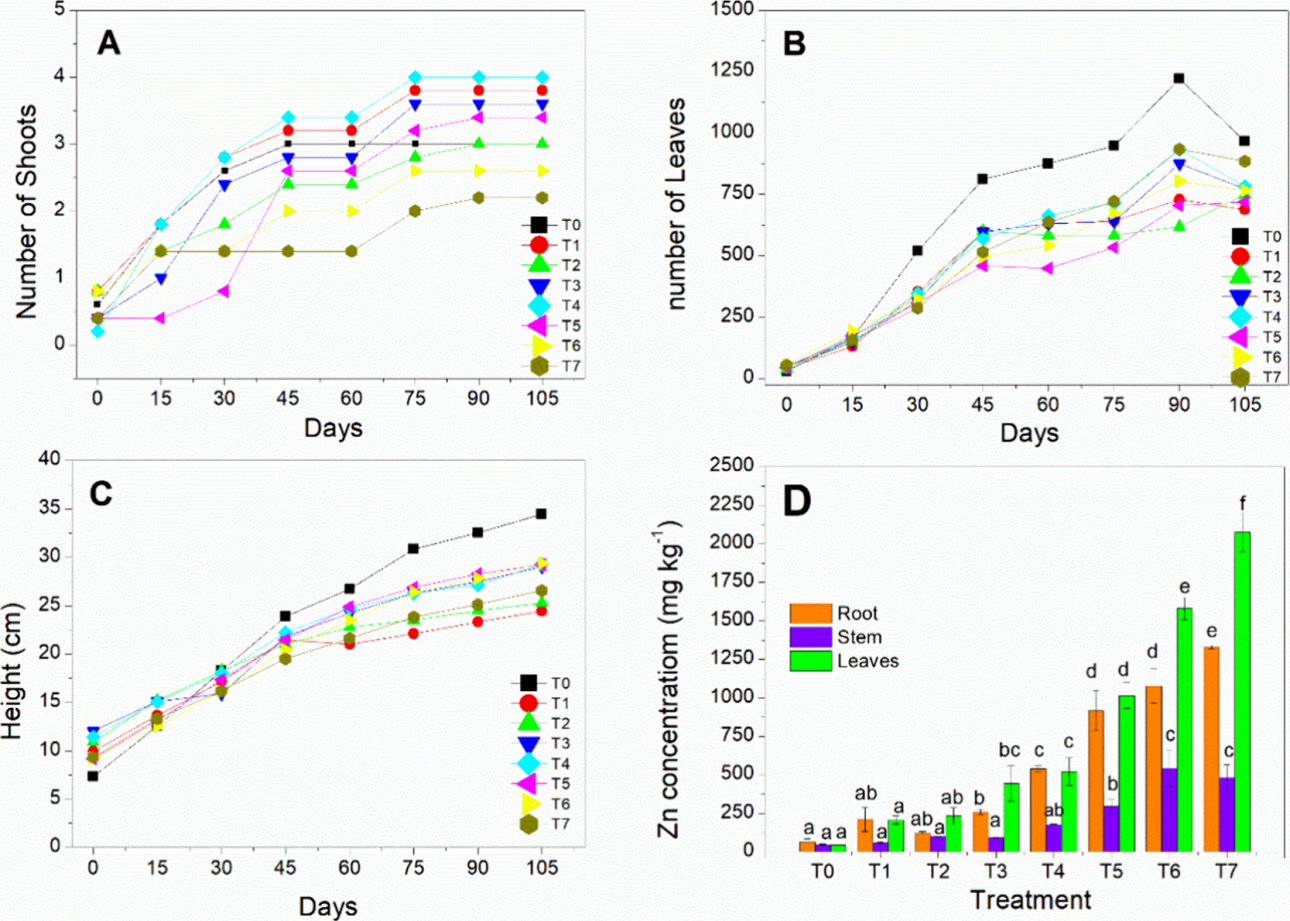
Growth of *Mentha crispa* cultivated for 105 days in Zn-contaminated soil, showing the average number of shoots (A), leaves (B), and height (C) for the applied treatments (T_1_ to T_7_) with T_0_ as the control. (D) Mean and standard deviation values for Zn concentration (mg kg^–1^, dry weight) in the roots, stems, and leaves under the same treatments. Equal letters indicate that the values are not significantly different between the treatments (Tukey’s Test, *p* < 0.05).

Regarding the number of shoots, leaves, and the height of the aboveground part over the 105-day experiment, the plant development can be seen in Figure 4A–C. Generally, with the increasing Zn concentrations in the soil, there was a trend of reduction in the number of leaves and plant height compared to the control treatment T_0_.

**Figure 5:**
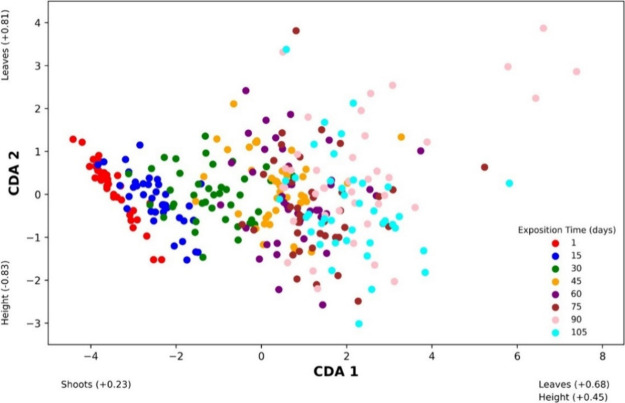
Correlation of the parameters: height, shoots, and leaves as a function of the 105-day cultivation period through Canonical Discriminant Analysis (Wilk’s Lambda = 0.19; *F* = 0.3491; *p* < 0.0001).

Canonical Discriminant Analysis (CDA) was applied to evaluate which variables (number of leaves, number of shoots, and height) associated with plant growth contributed most to the discrimination considering the time (days) of exposure to Zn (Figure 5). The interpretation of the analysis is based on the CDA 1 axis, which explains 98% of the data variability, demonstrating that the number of shoots was the most affected variable up to 30 days of the experiment. From 45 days onward, the data became more dispersed, particularly regarding the number of leaves (the most positive coefficient on the CDA 1 axis; Figure 5). The influence of plant exposure time to Zn on the number of leaves is consistent with Figure 4B, which showed a tendency for reduction in the treatments with Zn in the soil compared to the control treatment T_0_.

**Figure 6:**
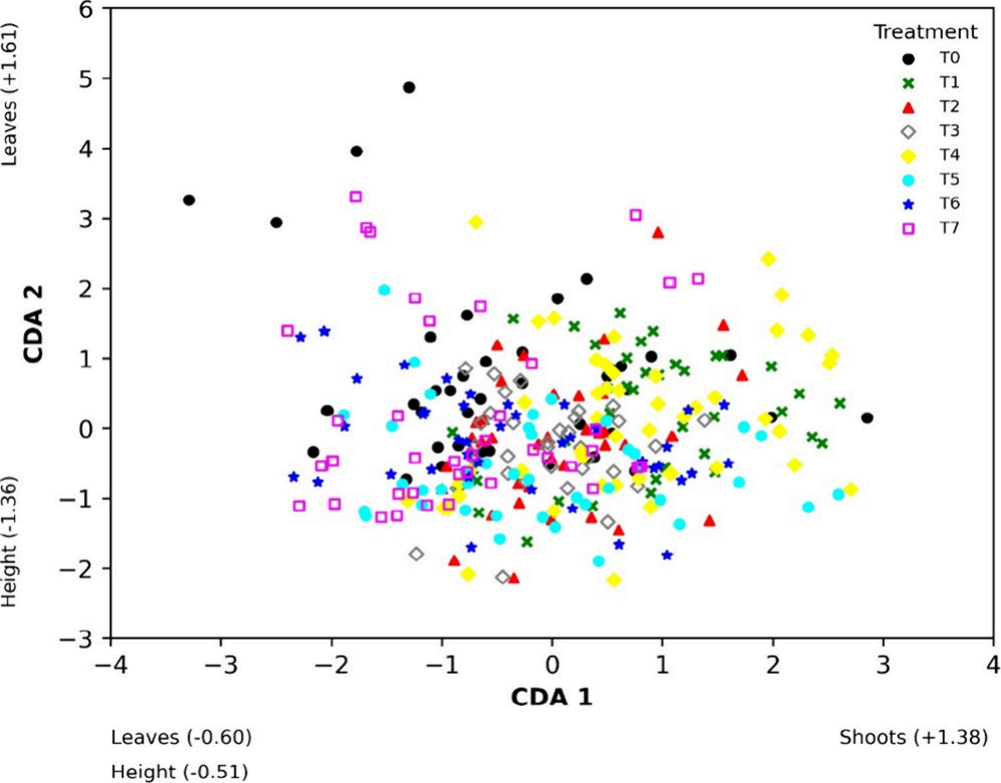
Scores of axes 1 and 2 of the Canonical Discriminant Analysis (CDA) for the growth variables responses of *Mentha crispa* (plant height, number of shoots and leaves) in the different Zn concentrations in the Oxisol (T_1_ to T_7_ treatments, and T_0_ as control). Values in parentheses correspond to the standardized coefficients of plant growth variables for the respective axes of the CDA (Wilk’s Lambda = 0.68; *F* = 6.03; *p* < 0.0001).

Considering the different Zn concentrations applied to the soil (treatments T_0_ to T_7_), the model obtained through CDA also identified the variables associated with plant growth that contributed most to the discrimination after the 105-day experiment (Figure 6).

The first canonical axis (CDA 1), explaining 62% of the variability, demonstrated that the number of shoots was the most influenced variable, showing the highest positive score on the CDA 1 axis (+1.37). This result is consistent with Figure 4A, as the number of shoots was the only growth parameter where the control treatment T_0_ did not differ from the other applied treatments. Therefore, the CDA suggests no significant difference in the number of shoots between the Zn-added treatments and the control treatment T_0_.

The second canonical axis (CDA 2) in Figure 6 explains 32% of the data variability, with the number of leaves being the most affected variable, showing the highest positive score (+1.64). The T_0_ treatment differed the most from the other treatments, consistent with the observations in Figure 4B regarding the number of leaves. Therefore, the CDA indicates a significant difference in the number of leaves between the Zn-added treatments and the control treatment T_0_.

### 3.4 Zn Concentration in the Roots, the Stems, and the Leaves

After 105 days of cultivation, the plants were harvested, and the Zn content was determined in the roots, stems, and leaves based on dry weight. The results are presented in Figure 4D. Generally, the Zn concentration was higher in the leaves and roots for all treatments except the control treatment T_0_. There was an increase in Zn absorption of approximately 28 times in the leaves, 20 times in the roots, and 10 times in the stems for treatment T_7_ compared to the control T_0_.

Similar results for Zn absorption were found by Lajayer et al.^62^ in the cultivation of *Mentha pulegium* in soil treated with 50 mg kg^–1^ of Zn, accumulating 204.74 mg kg^–1^ of the metal in the roots and 156.97 mg kg^–1^ in the leaves. In this study, considering the soil from treatment T_1_ (60 mg kg^–1^), *Mentha crispa* accumulated 213 mg kg^–1^ in the roots and 206 mg kg^–1^ in the leaves.

**Figure 7:**
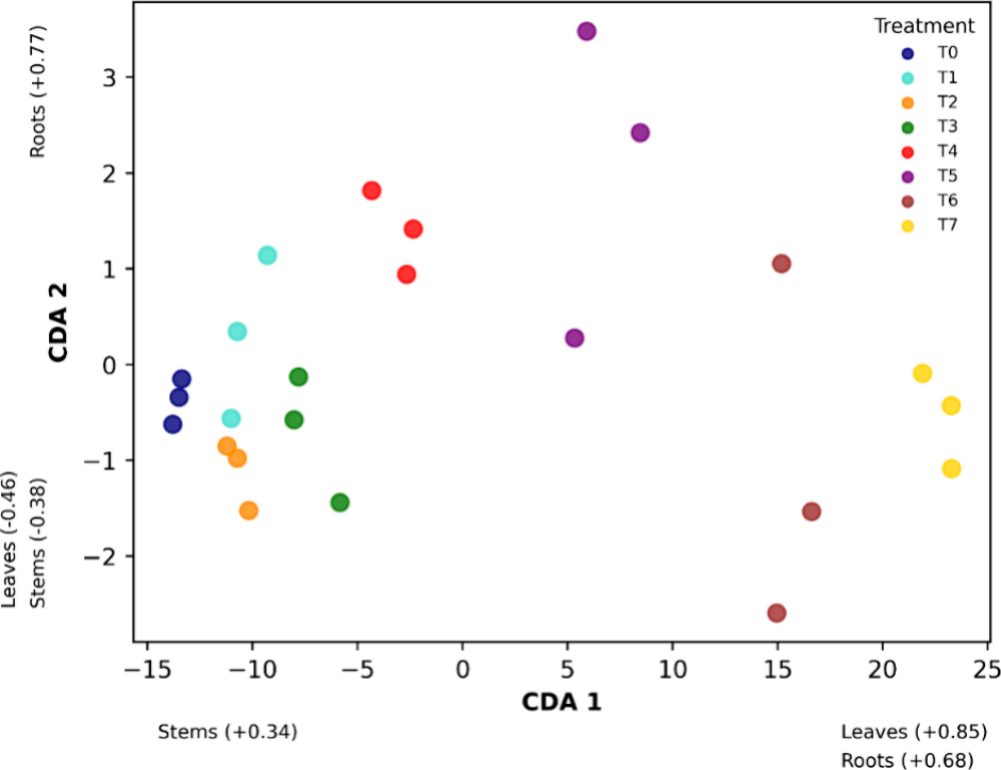
Scores of axes 1 and 2 of the canonical discriminant analysis for the Zn accumulation responses in the parts of the *Mentha crispa* (roots, stems, and leaves) in the different Zn concentrations in the Oxisol (T_1_ to T_7_ and T_0_ as control). Values in parentheses correspond to the standardized coefficients of body variables for the respective axes of the CDA. (Wilk’s lambda = 0.000730; *F* = 22.06; ρ < 0.001).

CDA was applied to verify if there was a significant difference in Zn absorption by the plant between the applied treatments and to identify which parts of the plant (roots, stems, and leaves) were most affected. The resulting graph can be seen in Figure 7.

The Wilk’s Lambda value of 0.0007303 demonstrates a strong discrimination between the treatment groups with different Zn concentrations. The associated ρ-value for Wilk’s Lambda test was <0.0001, which is highly significant and indicates a statistically significant difference between the treatment groups based on the Zn absorption variables.

The CDA 1 axis was significantly more important in ordering of results, explaining 99% of the data variability. According to this axis, the highest canonical coefficients were obtained for the variables leaves (+0.85) and roots (+0.68), indicating that these were the most affected by the Zn treatments. Figure 7 shows a clear separation between the treatments, suggesting that different Zn concentrations in the soil result in different patterns of metal absorption in the plant. Treatments with higher Zn concentrations (T_6_ and T_7_) are separated from those with lower concentrations (T_0_ to T_3_).

The significant increase in Zn concentration in the roots and leaves can be explained by the maximum adsorption capacity (MAC) of 456 mg kg^–1^ obtained from the Langmuir isotherm. The range of Zn concentrations applied in the treatments of this study considered the MAC to establish concentrations below and above this value. The Zn concentration in treatment T_5_ is very close to the MAC and the investigation threshold for agricultural areas of 450 mg kg^–1^ in Brazil.^11^ In treatments below T_5_, Zn species were more adsorbed by soil particles, making metal absorption by the plant more difficult. Treatments T_6_ and T_7_, with Zn concentrations above the MAC, had a higher fraction of free Zn^2+^ in the soil solution, providing greater availability of the metal to be absorbed by the plant.

Zn accumulation in plants can primarily occur in the leaves and roots, with higher concentrations in the aboveground parts, especially in hyperaccumulator plants.^10,63^ Toxicity generally manifests when the Zn concentration in leaves exceeds the range of 400–500 mg kg^–1^.^5,10,63,64^ The Zn concentration in the leaves of *Mentha crispa* for treatment T_7_ was approximately four times higher (1875 mg kg^–1^) than the concentration considered the threshold for toxicity, indicating that the plant is tolerant to high Zn concentrations in the soil.

Zn is absorbed by the roots primarily as Zn^2+^, but it can be transported as a complex with organic ligands to the leaves. The transport is carried out via the xylem through the cortex and epidermal cells, utilizing symplastic pathways (via plasmodesmata) and apoplastic pathways (outside the cells). Once in the xylem, Zn is translocated to the aerial parts of the plant.^5,10,63,65−67^

### 3.5 Bioaccumulation and Translocation Factors

The potential of *Mentha crispa* for zinc (Zn) removal from soil to its aerial parts was assessed through the Bioaccumulation Factor (BF), Translocation Factor (TF), Metal Extraction Rate (MER), number of vegetative cycles, and estimated removal time (Table 4).

**Table 4:** Factors of Bioaccumulation (BF) and Translocation (TF), Metal Extraction Rate (MER), Number of Vegetative Cycles, and Respective Zn Removal Time Estimate for *Mentha crispa* for Each Treatment (T_1_ to T_7_)

treatment	Zn applied in the soil mg kg^–1^	BF	TF	MER (%)	number of cycles^a^	estimated removal time (years)
T_1_	60	4.3	1.2	2.2	45	13
T_2_	80	4.2	2.7	1.8	56	16
T_3_	120	4.5	2.1	2.4	42	12
T_4_	240	3.3	1.5	1.8	56	16
T_5_	480	2.7	1.4	1.5	67	19
T_6_	960	1.8	1.6	1.0	100	29
T_7_	1920	1.2	1.8	0.8	119	34

a Vegetative cycle of the plant was considered 105 days.

Bioaccumulation Factor (BF) and Translocation Factor (TF) are key indicators in assessing a plant’s phytoremediation potential.^47,68^ BF reflects the plant’s capacity to absorb metals from the soil into its tissues, with values greater than 1 indicating efficient uptake. TF measures the movement of metals from the roots to the aerial parts (stems and leaves); a TF greater than 1 suggests effective translocation, making the plant suitable for phytoextraction. These factors help determine how well a plant can remove contaminants from soil and store them in harvestable parts for safe disposal.

The plant absorbs Zn through transporter proteins such as ZIP (Zinc–Iron Permease) and HMA (Heavy Metal ATPase), facilitating Zn transport from roots to aerial parts.^69,70^ These transporters regulate metal uptake and translocation, enabling accumulation in stems and leaves. However, soils like Oxisol, iron, and aluminum oxides firmly retain much of the Zn, reducing its availability to plants and directly impacting bioaccumulation and extraction values.^71,72^

The data in Table 4 offers a view of *Mentha crispa’s* behavior regarding Zn absorption, translocation, and extraction across different Zn concentrations in Oxisol. In treatments with lower Zn concentrations (T_1_ to T_3_ in Table 4), BF values were higher, ranging from 4.2 to 4.5, indicating efficient Zn uptake in these conditions. As soil Zn concentrations increased (T_4_ to T_7_), BF values declined to 1.2 in T_7_, which may be due to saturation of root Zn uptake mechanisms and strong metal retention by the soil, reducing its bioavailability. Additionally, Zn toxicity at higher concentrations may impair root function, decreasing water and nutrient uptake, further lowering BF. Figure 3B illustrates reduced root volume in treatments with higher soil Zn concentrations.

The Translocation Factor (TF) remained consistently above one (1) across all treatments, indicating that *Mentha crispa* effectively translocates Zn from roots to aerial parts, even at elevated soil Zn concentrations. In treatments, T_6_ and T_7_, slight increases in TF (1.6 and 1.8, respectively) suggest that the plant prioritizes metal transport to aerial parts as a defense mechanism against Zn toxicity in roots.^5^ This behavior is advantageous for phytoremediation, as accumulated Zn in aerial parts can be removed via harvest.

Although TF remained high, *Mentha crispa* did not reach the threshold of 3000 mg kg^−1^ Zn in aerial parts to qualify as a hyperaccumulator plant,^63,73,74^ reaching 2359 mg kg^−1^ in T_7_. These findings indicate that *Mentha crispa* can be classified as a Zn accumulator plant, with potential for phytoremediation in areas with moderate Zn contamination.

The Metal Extraction Rate (MER %),^48,68^ the number of cycles, and the estimated Zn removal time were calculated for each treatment (Table 4) considering only the Zn accumulated in the aerial parts (stems + leaves). Fresh biomass weights (see Supporting Information) and the 105-day vegetative cycle were used for these calculations.

The MER reveals that *Mentha crispa* exhibits light Zn extraction efficiency. In treatments T_1_ to T_3_, MER varied between 1.8 and 2.4%, indicating good Zn removal capability at lower soil concentrations. However, as Zn concentrations rise, MER decreases, reaching just 0.8% in T_7_, reflecting both limitations in Zn uptake due to saturation of transport mechanisms^5^ and Zn adsorption by Oxisol.^71^ Zn toxicity at higher concentrations also hinders root growth (Figure 3B), affecting metal uptake capacity.

MER did not exceed 2.4% for all treatments, requiring between 45 and 119 vegetative cycles. Soil with Zn contamination at 480 mg kg^–1^ (T_5_)–near Brazil’s investigation threshold for agricultural areas^11^–has an estimated removal time of 19 years. While removal times are relatively long, improved agricultural practices, such as enhancing soil quality, optimizing irrigation, applying fertilizers, and adjusting plant spacing, could increase biomass production and Zn extraction efficiency, potentially reducing remediation time.^75^

While conventional remediation methods, such as Pump-and-Treat or Soil Vapor Extraction, are faster, they are often prohibitively costly and environmentally disruptive, making them unsuitable for large-scale applications, especially in developing regions.^76^ In contrast, despite longer time scales, phytoremediation using *Mentha crispa* provides a low-cost, eco-friendly alternative for Zn removal. In contrast, despite longer time scales, phytoremediation using *Mentha crispa* provides a low-cost, eco-friendly alternative for Zn removal. Furthermore, the economic potential of essential oil production from *Mentha crispa* enhances its appeal. Steam distillation used to extract essential oils poses minimal risk of heavy metal contamination since these metals generally remain in the plant’s solid parts and do not volatilize during distillation.^17^

Although this study did not analyze essential oils, research on other *Mentha* species, such as *Mentha arvensis*, has shown that heavy metals like Cd and Pb do not significantly affect essential oil yield or composition.^33^ Future research will assess the composition and quality of essential oil extracted from *Mentha crispa* grown in metal-contaminated soils to determine the effects of Zn and other heavy metals.

Another critical aspect is using Oxisols as the substrate in this study, which plays a critical role in heavy metal adsorption. Oxisols, prevalent in tropical regions, are rich in iron and aluminum oxides, providing a high capacity for metal adsorption, such as Zn. Studies demonstrate that these soils effectively stabilize metal contaminants, reducing their bioavailability and minimizing adverse impacts on plants.^6,12,13^ The retention capacity of Oxisols is crucial for phytoremediation in tropical regions, limiting metal mobility in the soil and preventing leaching into groundwater.^6^ These findings underscore the potential of *Mentha crispa* for Zn phytoremediation, emphasizing the vital role of Oxisols in metal adsorption and stabilization, and the physiological adaptations enabling plants to thrive in contaminated environments.

This study demonstrates the potential of *Mentha crispa* as a sustainable and effective solution for remediating Zn-contaminated Oxisols. The plant showed high tolerance to Zn, with significant accumulation in leaves and roots, while maintaining normal growth. The bioaccumulation and translocation factors reinforce its capacity to absorb and distribute Zn efficiently, positioning it as a promising accumulator species.

In conclusion, *Mentha crispa* emerges as a viable and sustainable alternative for remediating Zn-contaminated Oxisols. This strategy integrates environmental recovery with economic opportunities, offering a practical solution for addressing soil contamination in tropical regions. Future research should focus on optimizing cultivation practices and assessing the quality of essential oils under varying contamination scenarios to maximize both environmental and economic benefits.

## Supporting Information Available

Additional details relevant to this study, including raw data from the experiments, supplementary tables and figures illustrating the growth and Zn accumulation in *Mentha crispa* under varying soil conditions and complement the findings presented in the manuscript and provide further insights into the experimental procedures and outcomes (PDF)
