# Supplementary material for: Phytoremediation Assessment of Mentha crispa L. in Zinc-Contaminated Oxisols: Tolerance and Accumulation Dynamics
Source: J Agric Food Chem. 2024 Dec 31;73(2):1086–96. doi: 10.1021/acs.jafc.4c08062 (PMC11741099; doi:10.1021/acs.jafc.4c08062)
Supplement: Supplementary file 1 — jf4c08062_si_001.pdf [file jf4c08062_si_001.pdf]

Phytoremediation Assessment of *Mentha crispa L.* in Zinc-Contaminated Oxisols: Tolerance and Accumulation Dynamics

Ana Flávia Bilmayer[1], Stephanie Locatelli[2], Martina Pomini[2], Thayná Francine Reis[2], Marcelo Hidemassa Anami[2], Edson Fontes de Oliveira[1,2], Robert Kowalik[3], Adriana Zemiani Challiol[4], Alessandra Furtado da Silva[1*]

*Correspondent author: alessandrasilva@utfpr.edu.br

Supporting Information

Fresh biomass of *Mentha crispa* plant parts for each treatment with different Zn concentrations in the soil.

Treatment	Stem (g)	Leaves (g)	Stem+Leaves (g)	Roots (g)
T_0	2.76	3.06	5.82	8.04
T_1	5.64	4.68	10.32	16.56
T_2	4.68	3.96	8.64	18.72
T_3	5.16	5.4	10.56	20.88
T_4	5.28	5.52	10.8	36.12
T_5	5.4	5.88	11.28	28.68
T_6	6.84	4.49	11.33	20.4
T_7	8.28	5.4	13.68	7.8

Zn concentrations determined by flame atomic absorption spectrometry in different parts of *Mentha crispa* (roots, stem, leaves, and stem + leaves) for each treatment.

Treatment	Zn Treatment (mg kg^{-1})	Roots (mg kg^{-1})	Stem (mg kg^{-1})	Leaves (mg kg^{-1})	Stem+Leaves (mg kg^{-1})
T_0	0	65.0	49.4	66.9	116.3
T_1	60	212.9	61.8	196.0	257.8
T_2	80	121.9	100.4	232.9	333.3
T_3	120	259.6	93.9	443.1	537.0
T_4	240	537.9	176.0	622.3	798.3
T_5	480	918.2	296.9	982.5	1279.4
T_6	960	1079.0	541.4	1149.5	1690.9
T_7	1920	1330.9	484.1	1875.4	2359.5

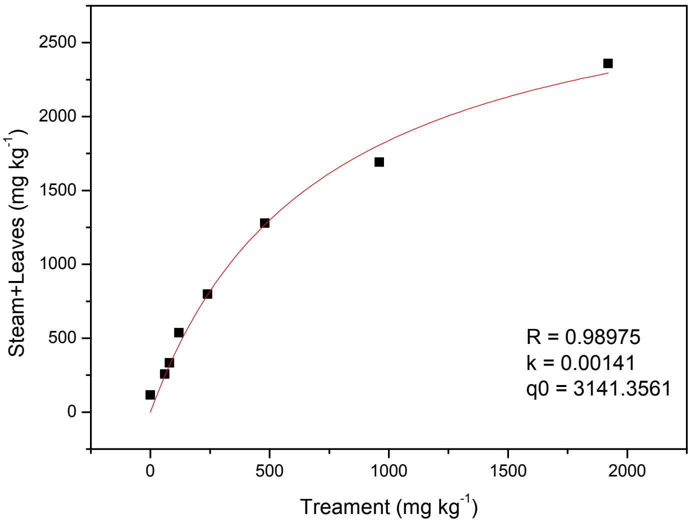

Langmuir isotherm showing Zn concentration absorbed by the aerial parts (stems + leaves) of *Mentha crispa* as a function of Zn concentration applied to the soil (T_0 to T_7 treatments).

Mean and standard deviations values of plant height (cm), number of leaves and number of shoots for *Mentha crispa* cultivated in Oxisol contaminated with Cd (treatments from T_0 to T_7) for 105 days of experiment. Scott-Knott test 5%.

	**Days**						
	**15**	**30**	**45**	**60**	**75**	**90**	**105**
				**Number of Leaves**			
T_0	165.8 ± 32.8 a	520.4 ± 77.7 a	811.2 ± 174.0 a	875.0 ± 162.5 a	947.8 ± 239.4 a	1520.0 ± 432.5 a	965.6 ± 262.9 a
T_1	131.0 ± 67.2 a	354.6 ± 75.0 a	597.8 ± 67.8 b	631.0 ± 80.6 b	641.4 ± 90.3 b	729.6 ± 65.8 b	687.0 ± 103.4 b
T_2	149.4 ± 37.7 a	335.2 ± 98.3 a	600 ± 71.0 b	582.8 ± 83.9 b	582 ± 108.3 b	617.8 ± 102.7 b	748.8 ± 172.3 b
T_3	148.2 ± 60.0 a	315.4 ± 174.2 a	598.4 ± 56.0 b	629.8 ± 36.5 b	640 ± 47.2 b	875.2 ± 70.1 c	772.2 ± 71.7 b
T_4	167.2 ± 21.1 a	346.6 ± 44.2 a	569.2 ± 51.5 b	663.8 ± 102.8 b	714.8 ± 103.9 b	932.6 ± 149.7 c	783.2 ± 133.5 b
T_5	175.0 ± 68.3 a	309 ± 122.2 a	458.8 ± 34.1 b	449.0 ± 66.7 c	534.4 ± 91.6 b	705.2 ± 210.2 b	718.6 ± 96.7 b
T_6	192.8 ± 95.0 a	324.6 ± 119.4 a	496 ± 120.9 b	542.0 ± 123.7 c	672 ± 125.2 b	804.0 ± 138.2 c	766.0 ± 129.7 b
T_7	156.8 ± 33.8 a	287.6 ± 91.7 a	514.8 ± 116.6 b	637.2 ± 144.4 b	721.4 ± 190.4 b	934.0 ± 227.5 c	884.4 ± 41.8 c
				**Number of Shoots**			
T_0	1.8 ± 1.5 a	2.6 ± 1.0 a	3.0 ± 1.0 a	3.0 ± 1.0 a	3.0 ± 1.0 a	3.2 ± 1.0 a	3.2 ± 1.0 a
T_1	1.8 ± 0.8 a	2.8 ± 0.8 a	3.2 ± 0.8 a	3.2 ± 0.8 a	3.8 ± 0.8 a	3.8 ± 0.8 a	3.8 ± 0.8 a
T_2	1.4 ± 0.8 a	1.8 ± 0.5 b	2.4 ± 0.5 a	2.4 ± 0.5 a	2.8 ± 0.8 a	3.0 ± 0.7 a	3.0 ± 0.7 a
T_3	1.0 ± 0.5 a	2.2 ± 0.4 a	2.7 ± 0.4 a	2.7 ± 0.4 a	3.2 ± 0.9 a	3.2 ± 0.9 a	3.2 ± 0.9 a

T_4	1.6 ± 1.3 a	2.8 ± 0.5 a	3.3 ± 0.5 a	3.3 ± 0.5 a	4.1 ± 0.7 a	4.1 ± 0.7 a	4.1 ± 0.7 a
T_5	0.4 ± 0.8 a	0.8 ± 1.1 b	2.6 ± 1.1 a	2.6 ± 1.1 a	3.2 ± 1.5 a	3.4 ± 1.1 a	3.4 ± 1.1 a
T_6	1.4 ± 1.5 a	1.4 ± 1.4 b	2.0 ± 1.4 b	2.0 ± 1.4 b	2.6 ± 1.1 a	2.6 ± 1.1 a	2.6 ± 1.1 a
T_7	1.4 ± 0.9 a	1.4 ± 0.9 b	1.4 ± 0.9 b	1.4 ± 0.9 b	2.0 ± 1.2 a	2.2 ± 1.1 a	2.2 ± 1.1 a
**Height, cm**							
T_0	7.3 ± 1.8 a	12.5 ± 2.6 a	18.2 ± 3.9 a	23.8 ± 4.0 a	26.7 ± 4.9 a	30.8 ± 5.1 a	34.5 ± 4.9 a
T_1	9.9 ± 2.8 a	13.6 ± 3.0 a	17.1 ± 4.0 a	21.4 ± 4.1 a	21.0 ± 3.9 a	22.1 ± 2.9 a	23.3 ± 3.1 b
T_2	10.9 ± 4.6 a	15.1 ± 3.5 a	18.2 ± 2.7 a	21.0 ± 2.7 a	22.7 ± 2.9 a	23.5 ± 3.1 a	24.4 ± 3.5 b
T_3	10.2 ± 5.0 a	13.6 ± 2.9 a	16.9 ± 2.8 a	22.9 ± 2.3 a	25.2 ± 2.8 a	27.5 ± 3.1 a	28.7 ± 3.3 b
T_4	12.7 ± 1.5 a	16.0 ± 1.7 a	17.0 ± 2.1 a	21.4 ± 2.8 a	23.9 ± 3.8 a	25.4 ± 4.7 a	26.3 ± 5.2 b
T_5	9.1 ± 3.7 a	13.1 ± 3.7 a	17.3 ± 3.1 a	21.4 ± 2.9 a	24.8 ± 2.4 a	26.9 ± 2.5 a	28.2 ± 2.5 b
T_6	9.2 ± 3.3 a	12.5 ± 4.6 a	16.1 ± 5.0 a	20.4 ± 6.6 a	23.5 ± 6.8 a	26.4 ± 7.1 a	27.8 ± 7.5 b
T_7	9.3 ± 0.8 a	13.3 ± 2.6 a	16.2 ± 4.3 a	19.5 ± 5.8 a	21.6 ± 7.2 a	23.8 ± 7.6 a	25.1 ± 8.7 b
